# Pulsed Electric Field Ablation of Esophageal Malignancies and Mitigating Damage to Smooth Muscle: An In Vitro Study

**DOI:** 10.3390/ijms24032854

**Published:** 2023-02-02

**Authors:** Emily Gudvangen, Uma Mangalanathan, Iurii Semenov, Allen S. Kiester, Mark A. Keppler, Bennett L. Ibey, Joel N. Bixler, Andrei G. Pakhomov

**Affiliations:** 1 Frank Reidy Research Center for Bioelectrics, Old Dominion University, Norfolk, VA 23508, USA; 2 Bioeffects Division, Airman System Directorate, 711th Human Performance Wing, Air Force Research Laboratory, JBSA Fort Sam Houston, San Antonio, TX 78234, USA; 3 SAIC, San Antonio, TX 78234, USA

**Keywords:** Barrett’s esophagus, electroporation, electropermeabilization, nanosecond pulses, IRE

## Abstract

Cancer ablation therapies aim to be efficient while minimizing damage to healthy tissues. Nanosecond pulsed electric field (nsPEF) is a promising ablation modality because of its selectivity against certain cell types and reduced neuromuscular effects. We compared cell killing efficiency by PEF (100 pulses, 200 ns–10 µs duration, 10 Hz) in a panel of human esophageal cells (normal and pre-malignant epithelial and smooth muscle). Normal epithelial cells were less sensitive than the pre-malignant ones to unipolar PEF (15–20% higher LD50, *p* < 0.05). Smooth muscle cells (SMC) oriented randomly in the electric field were more sensitive, with 30–40% lower LD50 (*p* < 0.01). Trains of ten, 300-ns pulses at 10 kV/cm caused twofold weaker electroporative uptake of YO-PRO-1 dye in normal epithelial cells than in either pre-malignant cells or in SMC oriented perpendicularly to the field. Aligning SMC with the field reduced the dye uptake fourfold, along with a twofold reduction in Ca^2+^ transients. A 300-ns pulse induced a twofold smaller transmembrane potential in cells aligned with the field, making them less vulnerable to electroporation. We infer that damage to SMC from nsPEF ablation of esophageal malignancies can be minimized by applying the electric field parallel to the predominant SMC orientation.

## 1. Introduction

Gastroesophageal reflux disease (GERD) affects more than 3 million people every year in the U.S. alone. Chronic exposure of the esophageal tissue to stomach acid, the prevalent symptom of GERD, can cause repeated damage to these cells. The recurrent injury to the lining of the esophagus can result in the development of Barrett’s esophagus, abnormal esophageal tissue lesions within the esophagus. Barrett’s esophagus is a recognized risk factor for developing esophageal adenocarcinoma, an aggressive cancer with a poor 5-year survival rate of approximately 15% [1,2,3]. Although Barrett’s esophagus does not in itself indicate a presence of pre-malignant tissue, a diagnosis requires close monitoring for progression of dysplasia, a pre-malignancy which can then develop into esophageal cancer [4]. Treatment options include ablation therapy, or, in the more extreme cases, endoscopic resection or esophagectomy. Ablation treatments are often used because they offer a less invasive option to target the pre-malignant lesions and prevent progression to esophageal cancer or an elimination method for already developed cancerous tissue. There are a variety of ablation modalities used in Barrett’s esophagus including photodynamic therapy, cryoablation, and radiofrequency ablation. Due to the adverse effects, lower efficacy and higher costs of photodynamic therapy, radiofrequency and cryoablation are more widely used options [4,5,6]. However, these procedures rely on the exposure of the tissue to extreme temperatures for cell death to occur, and often result in damage to the surrounding healthy tissues and further complications [7,8]. The need for new and improved techniques has opened a window for pulsed electric field (PEF) ablation. 

At high enough electric field strengths, PEF disrupts the cell membrane by a process known as electroporation or electropermeabilization [9,10,11]. PEF treatments that induce irreversible damage resulting in cell death have been proven to be a safe and effective method for eliminating cancers as well as benign hyperplasia [12,13,14,15,16,17,18,19,20]. PEF only affects cell membranes and therefore spares extracellular structural components such as collagen scaffolds and blood vessels, providing the framework for the repopulation of healthy, functional tissue. PEF ablation does not depend on the circulatory heat or cold dissipation, thus making it a tool of choice for complete removal of unresectable tumors located near large blood vessels [21,22]. In contrast, heat-dependent ablation techniques are limited in their applications in close proximity to major structures such as blood vessels and ducts [23].

Nerve excitation, which can cause pain and muscle contraction, is an undesirable side effect of PEF ablation. This side effect can be minimized with the use of shorter nano- and microsecond duration pulses [24,25,26,27,28], giving them a distinct advantage over the traditionally used 100 µs–1 ms “long” pulses. Further dampening of nerve excitation can be achieved with the use of bipolar pulses [24,25,26,27,28,29,30,31]. The electric field reversal increases the electric field thresholds for neuromuscular excitation by nano- and shorter microsecond-range pulses [32,33]. Bipolar pulses are also less efficient at electroporation and cell killing, yet the difference in cell killing is smaller than the increase in nerve excitation thresholds [24,25,26]. This makes short bipolar pulses the preference for PEF ablation with minimal neuromuscular side effects. 

The cell type plays a large role in the susceptibility of the targeted tissue as well as the surrounding healthy tissue in PEF treatments, and more research is needed to determine how to mitigate the damage to healthy tissues. The sensitivity to PEF treatments differs broadly across different cell types [34,35,36,37,38,39], which can work to the advantage of the treatment, or be a disadvantage. The potential to target specific cell types and spare the surrounding healthy tissues with a non-thermal method makes PEF an attractive modality for tumor ablation and cardiac ablation as well [40,41]. 

Due to the high importance of the structural tissue in the esophagus, the ability of PEF to spare extracellular tissue makes it an appealing treatment for Barrett’s esophagus that has progressed to dysplasia. In this study, we assessed the susceptibility of pre-malignant Barrett’s esophagus cells, normal esophageal epithelium, and esophageal smooth muscle cells to PEF of various pulse durations and pulse shapes. We found pre-malignant epithelium to be more susceptible to unipolar PEF exposures in comparison to healthy epithelium across all tested pulse durations (200 ns–10 µs). The sensitivity of smooth muscle cells (SMC), which are spindle-shaped, was determined by their orientation in the electric field in a pulse duration-dependent manner. Nanosecond-range pulses caused a larger change in the induced transmembrane potential and more cell damage when applied crosswise to a cell, while cell alignment with the electric field had a protective effect. This difference faded for longer pulses, in agreement with recent findings in cardiac cells [42]. We infer that damage to the smooth muscle layer in nsPEF ablation treatments, such as for Barrett’s esophagus, can be minimized by applying the electric field parallel to the predominant orientation of the cells. 

## 2. Results

### 2.1. Electric Field LD50 for PEF Ablation with Uni- and Bi-Polar Pulses

**Figure 1:**
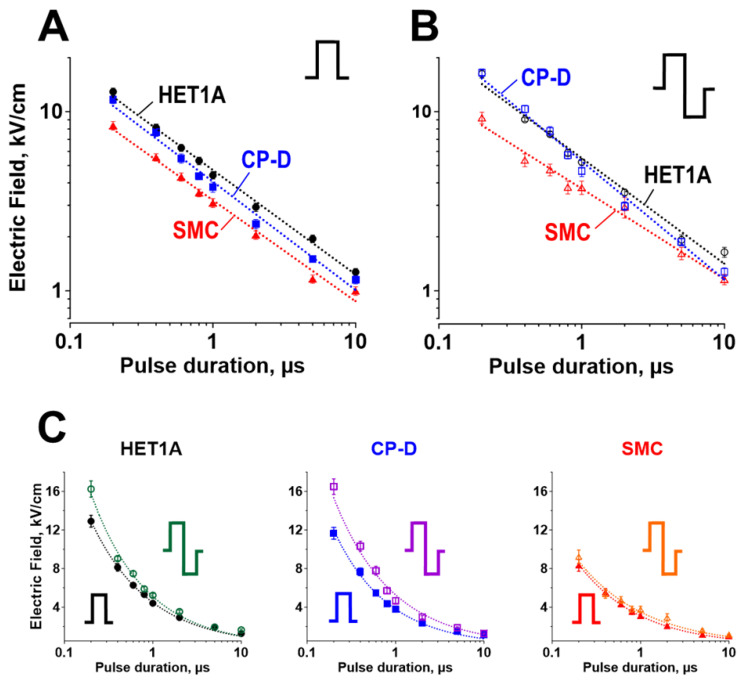
LD50 electric field strength for normal epithelial (HET1A), pre-malignant epithelial (CP-D), and smooth muscle cells (SMC) as a function of pulse duration for a 10 Hz, 100-pulse train. (**A**,**B**) display LD50 for uni- and bipolar pulses, respectively. (**C**) compares LD50 for uni- and bipolar pulses (filled and open symbols) in each individual cell type. For bipolar pulses in (**B**,**C**), the duration of one phase was used instead of the total pulse duration. Note a double-log scale in (**A**,**B**), but a semi-log scale in (**C**). For each individual data point, LD50 was determined by Hill equation fitting of cell death data from 5 to 14 independent experiments (see Section 4.4). Error bars (95% confidence intervals of the Hill fit at 50% survival) may not be visible if they are smaller than the central symbol. Dotted lines are the best fits of LD50 values using a power function.

Monolayers of three esophageal cell types (normal and pre-malignant epithelium and SMC) were exposed to trains of 100 uni- or bipolar pulses, at 10 Hz, with varying pulse durations and electric field strengths. The ratios of dead cells to the total number of cells were plotted against the electric field intensity (further explained in Section 4.4) from which the LD50 was extracted and plotted in Figure 1 for each pulse duration. LD50 values in the three cell types were compared across pulse (phase) durations from 200 ns to 10 µs, for uni-and bipolar pulses (Figure 1A,B, respectively). Figure 1C compares unipolar to bipolar LD50 values in each cell line to highlight the reduced efficiency of bipolar pulses and their dependence on the pulse duration. 

Normal epithelial cells were the least sensitive to unipolar PEF across all studied pulse durations, maintaining a 10–15% higher LD50 compared to the pre-malignant cells (*p* < 0.05 for most datapoints). SMC were the most sensitive to PEF, with LD50 about 20% lower than in pre-malignant epithelial cells (*p* < 0.05). These data indicate that unipolar pulses would likely cause relatively little off-target damage to normal epithelial cells but could impact nearby smooth muscle cells. 

Although bipolar pulses deliver twice the energy of unipolar pulses, they are typically less efficient at stimulation and electroporation [24,25,30,32,43]. The addition of the second phase increased the LD50 in both epithelial cell lines (by 15–20% for the shortest pulses, *p* < 0.01), but not in smooth muscle cells (Figure 1C). The difference in LD50 between normal and pre-malignant epithelial cells observed for unipolar pulses was no longer seen with bipolar pulses (Figure 1B). These results suggest that using bipolar pulses for ablation may eliminate the selectivity against cancer and worsen the off-target damage to smooth muscle. It remains to be seen if these disadvantages outweigh the reduced neuromuscular side effects of bipolar pulses [24,25,26].

**Figure 2:**
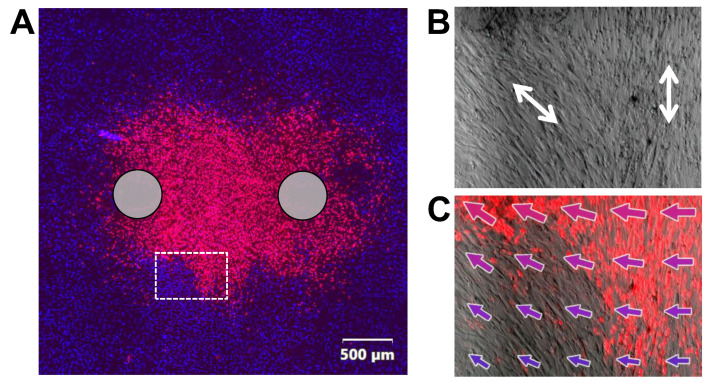
Cell survival depends on orientation in the electric field. (**A**) A non-oval cell death region (red, Pr staining) in a dense monolayer of smooth muscle cells (~100% confluency). Nuclei of live cells are counterstained blue with Hoechst dye. Cells were killed by applying 100 unipolar 600-ns pulses at 2.7 kV, 10 Hz between two electrodes (gray circles). The region outlined by a white rectangle is magnified in (**B**,**C**). (**B**) shows the bright field image of the monolayer, with the predominant cell orientation marked by white arrows. (**C**) shows the same bright field image overlayed with the Pr staining and electric field vectors. Vector lengths are proportional to the electric field strength. Cells oriented perpendicular to electric field are more likely to die, even at regions of weaker electric field.

In cell monolayer experiments summarized for Figure 1, lesion shapes were oval-shaped, following the electric field distribution (Section 4.8). Concurrently, we noticed that pulse treatment of overgrown SMC monolayers (~100% confluency; not used for Figure 1) produced irregular lesion shapes, particularly with pulse durations less than 1 µs (Figure 2A). Tightly packed, spindle-shaped SMC aligned with their neighbors, creating patches of cells oriented in the same direction. In contrast, epithelial cells stayed randomly shaped even at high confluency; they did not form patches of aligned cells or “protrusions” of the cell death regions. These observations suggested that there is a connection between SMC cell death and their orientation with respect to the electric field. Indeed, in patches where SMC were oriented parallel to the electric field, they survived better than in patches oriented perpendicular to the field (Figure 2B,C). Cells aligned parallel to the field survived electric field strengths which exceeded the lethal value for perpendicularly aligned cells.

### 2.2. Permeabilization with PEF in Different Cell Types

Permeabilization of individual cells by trains of ten, 300-ns unipolar pulses at 10 kV/cm, 5 Hz was measured by time lapse fluorescence imaging of YO-PRO-1 (YP) dye uptake. Images were collected every 10 s for 10 min, starting 45 s prior to nsPEF. The electric field was oriented across the long axis of SMC (within a +/− 30° angle), to emulate the worst-case scenario for the off-target tissue. Epithelial cells had irregular shapes and were not aligned with the field. 

**Figure 3:**
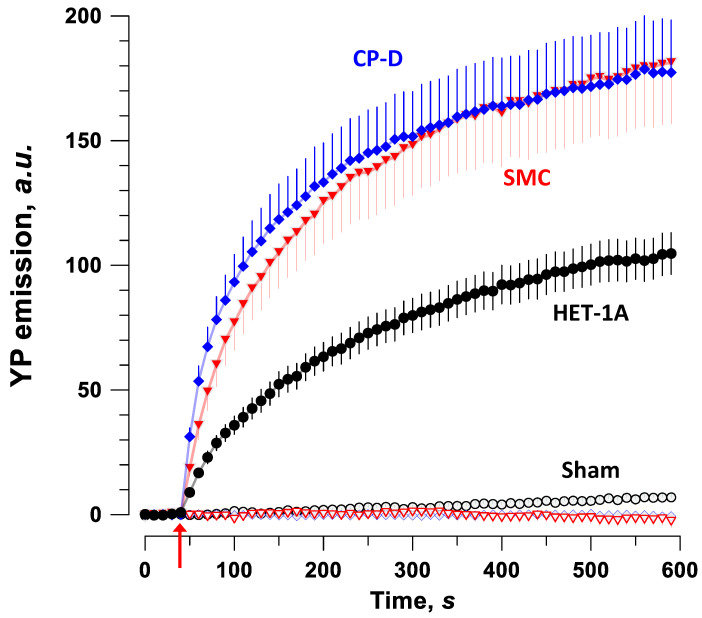
Electroporation of pre-malignant epithelium (CP-D), normal epithelium (HET-1A), and smooth muscle cells (SMC) measured by YP uptake after ten, 300-ns, 10 kV/cm pulses applied at 5 Hz (arrow). Spindle-shaped SMC were aligned perpendicular to the electric field; irregular-shaped epithelial cells were not oriented in the field. SMC and CP-D cells showed similar YP uptake throughout the 10-min recording. YP uptake in HET-1A was significantly lower, indicating a higher resistivity to permeabilization with electric pulses. Sham-exposed cells (open symbols) showed no YP uptake. Mean ± SEM, n = 19–40.

Similar to the LD50 measurements in cell monolayers, healthy epithelial cells were the most resistant to PEF, with about twofold less electroporative YP uptake than in either SMC or pre-malignant epithelial cells (Figure 3). The latter two cell lines showed nearly identical amplitude and time course of YP fluorescence gain due to the electroporative entry of YP, which contrasted with the relatively poor survival in SMC (Figure 1A). This result means that the extent of membrane disruption is not the sole predictor of cell survival and suggests an inferior SMC ability to recover from nsPEF-induced injuries. 

### 2.3. Permeabilization of SMC by Nanosecond Pulses Is Minimized by Aligning Cells with the Electric Field

**Figure 4:**
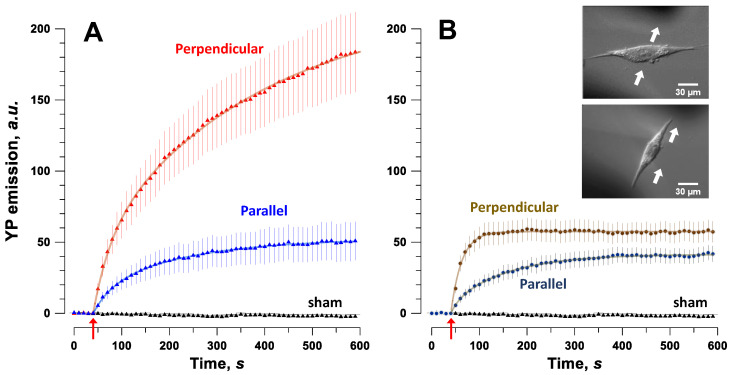
Smooth muscle cell orientation affects cell permeabilization with nanosecond pulses (**A**) more than with microsecond pulses (**B**). Permeabilization was measured by YP dye emission change following PEF treatment (arrow). (**A**) Cells were permeabilized by a train of ten, 300-ns pulses at 10 kV/cm, 5 Hz. (**B**) Cells were permeabilized by a train of ten, 20-µs pulses at 0.68 kV/cm, 5 Hz. Mean ± SEM, n = 12–17. YP uptake was significantly higher (*p* < 0.01) throughout the observation period (**A**), or only during the first minute (*p* < 0.05, (**B**)). Insets show an SMC between two electrodes (shadows) oriented either perpendicular or parallel to the electric field (top and bottom, respectively; arrows show the electric field direction).

These experiments were aimed at measuring how SMC alignment with the electric field affects membrane permeabilization. YP uptake was measured in different cell orientations for two pulse durations, 300 ns and 20 µs. Cells oriented approximately (within a +/− 30° angle) perpendicular or parallel to the electric field (Figure 4, insets) were exposed to ten pulses at 5 Hz of either 300-ns duration at 10 kV/cm, or 20-µs duration at 0.7 kV/cm. For 300-ns pulses, YP uptake in cells oriented perpendicular to the electric field was up to fourfold higher (*p* < 0.01) than in cells aligned with the field (Figure 4A). This difference was only twofold or less for 20-µs pulses but still statistically significant (*p* < 0.05) during the first minute after exposure (Figure 4B). This reduced difference in vulnerability between two cell orientations for longer pulses is an agreement with a recent modeling and experimental study in cardiac cells [42].

**Figure 5:**
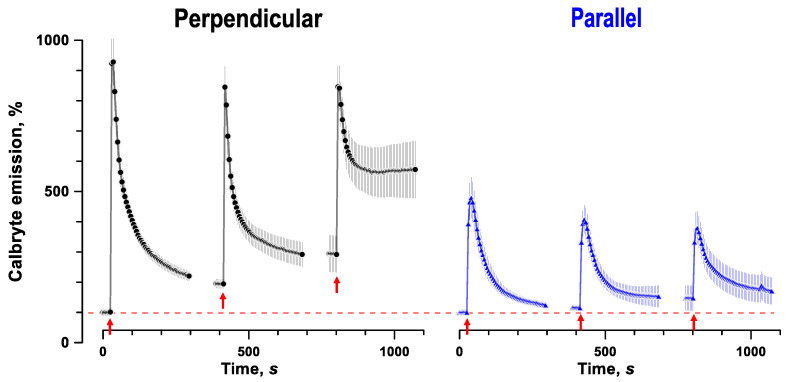
Calcium transients evoked by 300-ns, 14 kV/cm pulses in smooth muscle cells oriented perpendicular or parallel to the electric field (left and right panels, respectively). Each cell was stimulated three times (arrows). Note stronger response and impaired recovery to the baseline in cells oriented perpendicular to the field. Mean ± SEM, n = 12.

With more sensitive membrane permeabilization assays, such as imaging of cytosolic Ca^2+^ transients [44,45], higher vulnerability of SMC oriented perpendicular to the electric field can be revealed even with a single pulse (Figure 5). When cells loaded with a Calbryte Ca^2+^ indicator were stimulated by a 300-ns pulse at 14 kV/cm, dye emission increased nearly tenfold and fivefold in cells oriented perpendicular and parallel to the field, respectively. Evoked Ca^2+^ transients were likely a mixed effect of electroporation, activation of voltage-gated Ca^2+^ channels, and Ca^2+^-induced Ca^2+^ release from the endoplasmic reticulum. However, the inability of cells to restore the baseline Ca^2+^ level within minutes after stimulation was a clear indication of membrane damage. This damage was augmented by applying additional stimuli, resulting in recovery failure in cells oriented perpendicular to the field, but only a modestly slower recovery in cells aligned with the field. 

### 2.4. Cell Alignment Impacts Membrane Charging and Relaxation of the Induced Membrane Potential

**Figure 6:**
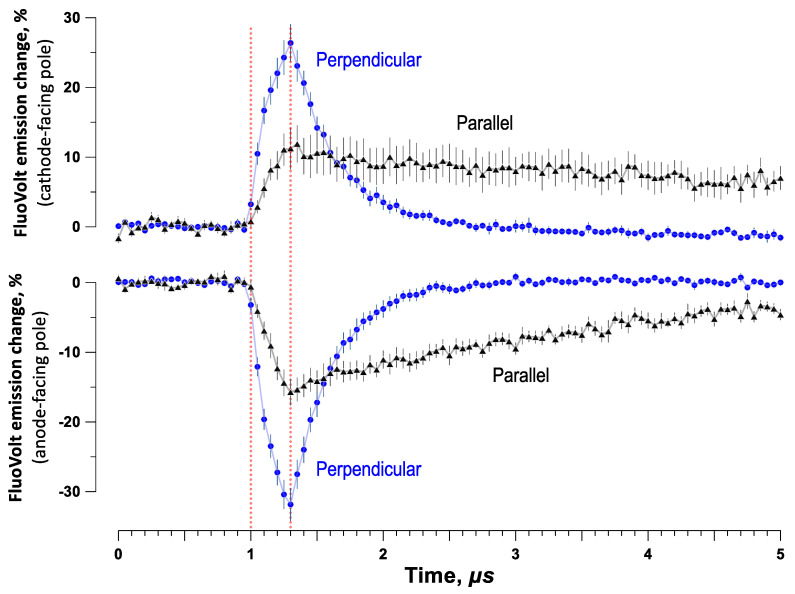
Membrane charging by a 300 ns, 0.3 kV/cm pulse is less efficient in smooth muscle cells aligned parallel with the electric field. The time course of the induced membrane potential was measured by strobe photography with a voltage sensitive FluoVolt dye and expressed as a percent change in the dye emission (mean ± SEM, n = 7). Vertical dotted lines denote the time when the pulse was applied. See text for more details.

Differences in nsPEF susceptibility in SMC aligned differently to the electric field prompted an investigation into the alignment impact on membrane charging. Time kinetics of charging by 300-ns pulses and relaxation of the induced membrane potential was resolved by strobe imaging with a voltage-sensitive FluoVolt dye [46,47]. These experiments focused on the passive membrane response, so the electric field intensity was set low at 0.3 kV/cm (below the electroporation threshold, which is at 1–2 kV/cm for 200–600 ns pulses [45,48,49]). The emission intensity of FluoVolt increased during the pulse at the cathode-facing side of the cell (depolarization) and decreased at the anode-facing side (hyperpolarization). Figure 6 shows time kinetics of the induced transmembrane potential (expressed as % change of FluoVolt emission) at the anode- and cathode-facing sides of cells oriented parallel and perpendicular to the field. The induced potential was twofold larger (*p* < 0.01) when the long axis of the cell was perpendicular to the electric field. Charging the cell membrane to a significantly higher potential explains stronger electroporation and poorer survival in cells oriented perpendicularly to the field. 

## 3. Discussion

This study explored the use of PEF ablative treatments on pre-malignant cells of Barrett’s esophagus and investigated potential damage to off-target tissues. The aggressive nature of esophageal cancer and poor survival rates highlight the need for non-invasive treatment options for the recognized precursor. Our study has shown that pre-malignant esophageal epithelium is more sensitive than normal epithelium to electroporation by PEF over a wide range of pulse durations, giving PEF another advantage over currently used ablation techniques. Changing pulse duration had little to no impact on the comparative sensitivity of pre-malignant and normal cells. While using nanosecond-range PEF would be neither advantageous nor disadvantageous for the selectivity against hyperplasia, it will cause less neuromuscular side effects than ablation treatments with longer pulses [24,25]. However, ablation with bipolar nsPEF (which would reduce neuromuscular effects even further) may eliminate the difference in PEF sensitivity between the normal and pre-cancer epithelium. Thus, the choice between using uni- or bipolar nsPEF for esophageal ablation treatments will depend on whether the advantage of further reduction in neuromuscular effects outweighs inflicting more damage to the normal epithelium.

Experiments that tested the survival of cells in a monolayer (Figure 1) showed that smooth muscle was the most sensitive to PEF treatments, followed by the pre-malignant cells and then the healthy epithelium. The permeabilization data, on the other hand, showed similar permeabilization and rate of fluorescent dye uptake between the smooth muscle and pre-malignant cells (Figure 3). This result suggests that the degree of membrane damage is not the sole predictor of cell survival, and that damaged SMC are less capable of recovering from PEF-induced damage than similarly damaged pre-malignant epithelial cells. The exact mechanisms responsible for the poorer survival of SMC are not known, but may include less efficient membrane repair [50] and the reduced capacity to restore the cytosolic Ca^2+^ level and osmotic balance [35,49,51]. 

While studying the ablation area in SMC monolayers, we observed projections and indentations (Figure 2) indicating that the region of cell death was not determined by the electric field strength alone. These projections occurred in confluent monolayers and were larger and more frequent for nanosecond pulse treatments. Confluency forced the adjacent cells to maintain the same orientation, and we found that cells survive better in areas where they are oriented parallel to the electric field. The critical role of SMC orientation with respect to nsPEF was validated for diverse pulse parameters in experiments measuring electroporative YP dye uptake, cytosolic Ca^2+^ mobilization and clearance, and membrane charging kinetics. SMC oriented perpendicular to the field consistently experienced stronger effects of nsPEF, which is in stark contrast to expectations based on cell membrane charging by conventional electroporation pulses (0.1–10 ms). It is well known that the membrane potential reached at the electrode-facing poles of a round cell, or a lipid vesicle exposed to a conventional electric pulse is linearly proportional to the cell diameter [10,11]. Therefore, larger, round cells reach the electroporation threshold with a weaker imposed electric field and will likely suffer more damage than smaller round cells when the electric field is the same. This logic can be extended to elongated cells, where the distance between the electrode-facing poles of the cell (i.e., the distance in the direction of the electric field) determines the membrane potential induced by this field. Indeed, diverse elongated cells aligned with the electric field were more vulnerable to 10-ms [52], 50- and 200-µs [53], and 1- and 10-ms pulses [42]. However, in this study we observed exactly the opposite dependence on orientation for cells electroporated by 300- and 600-ns pulses (Figure 2, Figure 4, Figure 5 and Figure 6). Stronger effects in cells oriented perpendicular to the field persisted even with longer 20-µs pulses, although less pronounced (Figure 4B).

One possible explanation to this paradox is that the cell membrane does not get fully charged during a short, nanosecond-range pulse (consistent with Figure 6), and the charging rate rather than the steady-state charging ceiling determines the membrane potential reached. This situation is analogous to comparing the charging process of large vesicles (cells) with small vesicles (organelles) by nsPEF: small vesicles have a smaller charging time constant, charge faster, and for pulse durations comparable to the time constant, they reach a higher induced membrane potential than larger, slowly-charging vesicles [54,55,56]. This hypothesis is consistent with the observations that electroporation with longer 20-µs pulses was less dependent on cell orientation (Figure 4B), or was independent of the orientation [42]. However, this hypothesis hinges on the assumption that the charging time constant for spindle-shaped cells depends on their orientation in the electric field, which has not been tested. We plan to test this assumption directly, by performing additional strobe imaging studies and using pulses long enough for exponential fitting and extraction of the time constant of the charging process. If confirmed, this hypothesis should hold true not just for SMC but for a variety of elongated cells, because membrane charging on the sub-microsecond time scale is faster than any active responses and thus it will be unlikely to depend on cell physiology.

Manipulation of cell killing efficiency by placing electrodes in a certain alignment with cells in tissue adds important versatility to PEF-based ablation methods. This versatility can be employed to enhance the impact on the target tissue and to reduce unwanted off-site damage to healthy tissues. This versatility is a novel quality that further distinguishes PEF from diverse heating- and cooling-based ablation methods [6,7,8]; however, its utility for cancer ablation remains to be validated in animal and human studies. 

## 4. Materials and Methods

### 4.1. Cell Lines and Maintenance

Human esophageal epithelial cells (CRL-2692) and pre-malignant esophageal epithelial cells (CRL-4030, high-grade dysplasia cell line) were purchased from American Type Culture Collection (ATCC, Manassas, VA, USA). Human primary esophageal SMC were purchased from Cell Biologics (Cell Biologics Inc., Chicago, IL, USA, H-6089). All cell types were maintained at 37 °C, 5% CO_2_ in their recommended medium. Normal epithelial cells were cultured in the BEGM BulletKit (Cat. # CC-3170, Lonza Biosciences, Walkersville, MD, USA) and pre-malignant epithelial cells in MCDB-153 (Cat. # M7403 Sigma-Aldrich, St. Louis, MO, USA). The gentamycin-amphotericin B mix was excluded from the BEGM kit as recommended by ATCC. MCDB-153 was supplemented with 0.4 µg/mL hydrocortisone, 8.4 µg/L cholera toxin, 20 mg/L adenine, 1.5 g/L sodium bicarbonate (all from Sigma-Aldrich, St. Louis, MO, USA), 140 µg/mL bovine pituitary extract (ScienCell Research Laboratories, Inc., Carlsbad, CA, USA), 20 ng/mL human epidermal growth factor, 1× ITS, 4 mM glutamine (all from Thermo Fisher Scientific, Waltham, MA, USA), and 5% fetal bovine serum (Atlanta Biologicals, Norcross, GA, USA). 100 I.U./mL penicillin and 0.1 μg/mL streptomycin (GIBCO, Gaithersburg, MD, USA) was added to the BEGM kit and the MCDB-153. SMC were cultured in the complete smooth muscle medium with supplement kit (Cat. # M2268, Cell Biologics Inc., Chicago, IL, USA). 

### 4.2. PEF Generators

Exposures of cell monolayers and strobe imaging (Section 4.3 and Section 4.7) were carried out with an EPULSUS-FPM4-7 generator (Energy Pulse Systems, Portugal) [32,57]. For permeabilization measurements in individual cells (Section 4.5 and Section 4.6), we used a Model 6040 mainframe with a 202H high voltage insert (Berkley Nucleonics Corporation, San Rafael, CA, USA). Pulse delivery was synchronized with time lapse-image acquisition with a TTL pulse protocol from a model 1322A Digidata board using Clampex 10 software (Molecular Devices, Foster City, CA, USA) [43,58].

Pulse shapes and amplitudes were continuously monitored with a TDS3052C oscilloscope (Tektronix, Beaverton, OR, USA) using either a P2301B high-voltage probe (Qingdao Hantek Electronic, Qingdao, China) or the EPULSUS voltage monitor.

### 4.3. PEF Efficiency for Cell Killing in Monolayers

Cells were seeded on 24-well plates (μ-Plate 24 Well Black, IBIDI, Gräfelfing, Germany) coated with 0.01 mg/mL fibronectin, 0.03 mg/mL bovine collagen type I, and 0.01 mg/mL bovine serum albumin (all from Thermo Fisher Scientific). 

Epithelial cells were grown for 3–6 days to ~90% confluency, refreshing media as needed. SMC monolayers used for LD50 analysis were grown to an ~80% confluency to ensure cells were oriented randomly, instead of aligning in clusters with neighbor cells. On some other occasions (Figure 2), SMC were grown to confluency. To prevent any interference on cell survival from the various media compositions used for propagating each cell line, electroporation treatments and imaging in all cell lines were performed in the same medium, DMEM without phenol red (Cat. #21063-029, GIBCO) [59]. A total of 10 min prior to experiments, the cell line-specific growth medium was removed and replaced with DMEM supplemented with 10% fetal bovine serum (Atlanta Biologicals), 25 mM HEPES to mitigate pH changes outside the incubator, and 1% agarose to limit cell detachment. Hoechst-33342 (Thermo Fisher Scientific), a cell-permeant dye, was added to the media at 2.25 μM to counterstain cell nuclei.

Electric pulses were delivered with two tungsten rod electrodes, 0.5-mm diameter, spaced 1.7-mm center-to-center. An automated electrode delivery system, built from a modified 3D printer [24,60], was utilized to position the electrodes in the center of each well, orthogonal to the monolayer and touching the bottom of the well, followed by a 40-s pause to deliver pulses. After the experiment, monolayers were imaged in the same sequence as they were exposed to PEF, and this pause helped to keep the time interval between pulse delivery and imaging the same for all wells. Pulsing parameters included durations ranging from 200 ns to 10 μs for unipolar waveforms, and from 200 ns to 10 μs per phase for bipolar waveforms, all applied as trains of 100 pulses at 10 Hz. Pulse repetition rate did not exceed 10 Hz for all experiments, to prevent a significant temperature build-up [24]. Pulse amplitude was tuned to create lesions of similar size across all pulse durations. For bipolar pulses, the second phase immediately followed the first one and was of the same amplitude and duration. Throughout this paper, bipolar pulses are referred to by the amplitude (and the respective electric field) and duration of the first phase (not by the peak-to-peak amplitude or the total pulse duration).

After pulse delivery, cells were returned to the incubator for 2 h before adding 75 μM propidium (Pr) iodide. Pr, a cell-impermeable fluorescent dye (Thermo Fisher Scientific), was used to label cells that had not recovered their membrane integrity after 2 h, at which point it was assumed that electroporation was irreversible. The dye was allowed to incubate for 20–30 min before imaging with an IX83 microscope, using CellSens version 4.1 software (Olympus America, Hamden, CT, USA) with an automated MS-2000 scanning stage (ASI, Eugene, OR, USA) and an X-Cite 110LED illuminator (Excelitas Technologies Corporation, Waltham, MA, USA). A high-resolution image of the monolayer in each well was switched together from nine images of adjacent areas, taken with a 10×, 0.38 NA objective, using DAPI and Cy3 filter cubes, and an ORCA-Flash4 sCMOS camera (Hamamatsu, Japan) [24].

### 4.4. Image Analysis of PEF Lesions in a Cell Monolayer

**Figure 7:**
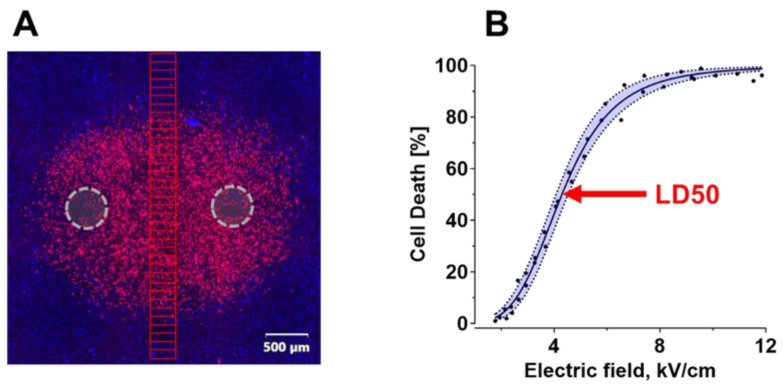
Calculation of LD50 from the fluorescent staining of a monolayer after PEF exposure. (**A**) A representative image of SMC monolayer labeled with Pr (red, dead cells) and Hoechst (blue, all cells; Hoechst staining in dead cells may be concealed by Pr). The monolayer was stained 2 h after applying 100, 600 ns pulses at 10 Hz between two electrodes (gray dashed line circles). Live and dead cells were counted in 36 ROI (red rectangles). (**B**) The percentage of dead cells in each ROI was plotted against the electric field in the center of the ROI and fitted using the Hill equation (solid black line); shaded area is a 95% confidence interval of the fit. Black dots are the mean cell death values from nine independent experiments.

Cell death regions were analyzed to find the electric field necessary to kill 50% of cells (LD50), following the procedures described recently [60]. In brief, 36 rectangular regions of interest (ROI), 300 µm × 100 µm each, were stacked and positioned directly between the electrode footprints, perpendicular to a line connecting the electrodes (Figure 7A). The electric field value assigned to each ROI was the value calculated by simulations (Section 4.8) for the center of that ROI. The number of dead cells (stained with Pr) was calculated as a percentage of all cells (stained with Hoechst) within each ROI using the cell-counting function in CellSens version 4.1 software. Cell death percentages were graphed against the electric field values for each ROI, and the data were fitted with a sigmoidal function utilizing Hill equation [61,62] (Figure 7B). The electric field value at which the sigmoidal function reached 50% was taken as the LD50 and graphed against the pulse duration. 

### 4.5. Electroporative Dye Uptake in Individual Cells

Single cell experiments were performed on an Olympus IX81 inverted microscope equipped with an FV1000 confocal laser scanning system (Olympus America, Center Valley, PA, USA) [43,58,63]. Cells were seeded 24–48 h prior to experiments on 12-mm glass coverslips (Neuvitro Co., Camas, WA, USA) pre-coated with either fibronectin (for epithelial cell lines) or gelatin (for smooth muscle cells). The coverslips with cells were rinsed with the physiological solution (140 mM NaCl, 5.4 mM KCl, 2 mM CaCl_2_, 1.5 mM MgCl_2_, 10 mM D-glucose, and 10 mM HEPES; 290–310 mOsm/kg, pH 7.4) and transferred into a glass-bottomed chamber (Warner Instruments, Hamden, CT, USA). The chamber was filled with the physiological solution supplemented with 1 µM of YO-PRO-1 dye (YP), a nucleic acid stain that is poorly permeant into healthy cells and is commonly used to measure electroporation [43,50,58,64]. 

Two tungsten rod electrodes (100 µm diameter, 150 µm gap) were positioned 50 µm above the surface of the coverslip, with a selected cell (or a small group of cells) centered between the tips using an MP-225 manipulator (Sutter Instruments, Novato, CA, USA). Pulse delivery was synchronized with image acquisition as described above in Section 4.2. Images of YP fluorescence (ex./em.: 488 nm/525–535 nm) were taken every 10 s for 10 min, beginning 45 s before PEF exposure. ROIs were manually drawn around the perimeter of each cell, and the fluorescence intensity was quantified with the ImageJ Fiji platform [65]. YP entry was quantified in arbitrary units as intensity (F) minus the baseline intensity (F0), an average of the first images taken before pulse delivery. Sham-exposed cells underwent all the same manipulations, but pulses were not delivered. Exposures using different pulse settings and sham exposures were alternated in a random manner. 

### 4.6. Ca^2+^ Transients in Smooth Muscle Cells

Cytosolic Ca^2+^ measurements in SMC were accomplished with the confocal microscope setup described above, using Ca^2+^ fluorescence indicator Calbryte (AAT Bioquest, Sunnyvale, CA, USA). Experiments followed the same procedures as described above in 4.5, except for loading cells with Calbryte immediately prior to experiments (20 min in the physiological solution with 2 μM of the dye). Images were taken every 5 s for 300 s, starting 25 s before pulse delivery. We used a single 300-ns pulse at 14 kV/cm and repeated the experiment three times in each cell (Figure 5). ROIs were manually drawn around the cell perimeter and average pixel intensity quantified with ImageJ. Intensity (F) was normalized to the baseline as (100% × F/F0), where the baseline intensity (F0) was a mean of the first images taken prior to the first pulse delivery. 

### 4.7. Kinetics of Membrane Charging and Discharging in SMC Exposed to Nanosecond PEF

Changes in the transmembrane potential (TMP) during and after the application of 300-ns electric pulses were recorded with a strobe imaging system described in detail recently [46,47]. Ultrashort laser pulses (~8 ns) were delivered in synchrony with nsPEF to excite FluoVolt dye, a voltage-sensitive fluorescent indicator. The camera shutter opened before nsPEF to capture a single fluorescence image at the moment of the laser flash, then closed. When the next nsPEF was delivered, the time interval between nsPEF and the laser flash was increased by 50 ns, and the next fluorescence image was captured. We delivered a total of to 200 nsPEF to build a time course of FluoVolt emission change during a 10-µs time interval that started 1 µs prior to nsPEF. (Figure 6 shows only the first 5 µs, to highlight charging kinetics during nsPEF). Strobe imaging experiments were aimed at studying the passive membrane properties (charging and discharging kinetics) not altered by any downstream biological effects. To avoid biological effects such as membrane rupture or opening of Ca^2+^ channels, strobe imaging was performed at a low electric field strength of 0.3 kV/cm and using short 300-ns pulses. The experiments could be done at any pulse repetition rate which allowed for complete membrane discharge between the pulses. We used the repetition rate of 6 Hz, which was the maximum rate the laser could fire, and it was much longer than needed for cell discharge. 

Our setup was modified from the original design [46] to use an Olympus IX71 microscope with an iXon Ultra 897 back-illuminated EMCCD Camera (Andor Technology, Ireland) and the EPULSUS generator. A customized q-switched neodymium-doped yttrium aluminum garnet (Nd:YAG) laser (Quantel USA, Bozeman, MT, USA) was synchronized with pulse delivery by custom made LabVIEW 2022 software utilizing a DG645 delay generator (Stanford Research Systems, Sunnyvale, CA, USA). A detailed description of the system was given in our recent publication [47].

Cells grown in a glass-bottomed 35-mm dish were loaded with FluoVolt diluted in the physiological solution (1/1000 FluoVolt stock and 1/100 PowerLoad Concentrate, Thermo Fisher Scientific) in the dark for 30 min. After loading, cells were rinsed, placed in fresh physiological solution, and transferred to the Olympus IX71 inverted microscope stage. 

Cells were exposed to pulses between two tungsten rod electrodes (0.5 mm diameter) placed 1 mm apart (side-to side). The electrodes were positioned in touch with the bottom of the dish and orthogonal to it with an MPC-200 manipulator (Sutter, Novato, CA, USA). The target cell was centered between the electrodes, either parallel or perpendicular to the electric field (with the angle accuracy within +/−20°). Each cell was tested in both alignments with the field, i.e., it was exposed to a total of 400 nsPEF in two strobe image experiments. The sequence of the experiments (i.e., which alignment was tested first) was varied randomly.

The intensity of the laser dye flash fluctuated within about +/−10%. The impact of this variability was minimized by scaling FluoVolt signal from the cathode- and anode-facing sides of the cell to the whole-cell FluoVolt signal (which is not affected by cell polarization in the electric field). The mean value for the datapoints collected during 1 µs before nsPEF was taken as 100%. Normalized emission data were averaged across the cell population for the different cell orientations in the electric field, and plotted as a percent difference from the initial value. 

### 4.8. Electric Field Simulations 

**Figure 8:**
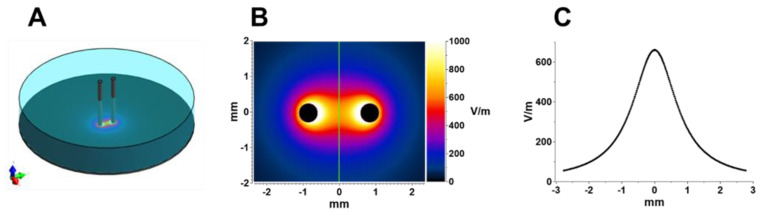
Electric field simulations performed with Sim4Life. (**A**): Electrode configuration model used for simulations. (**B**) Electric field values 5 µm above the surface of the dish for 1 V applied between the electrodes. (**C**) Electric field profile along the green line in panel (**B**).

Electric fields were calculated using a low-frequency finite element solver Sim4Life V5.2 (Zurich Med Tech, Zurich, Switzerland). For the experiments performed on a monolayer, two cylindrical rods (0.5 mm diameter, spaced 1.7 mm center-to-center) were inserted into a solution with a conductivity of 1.4 S/m. The parallel rods were positioned orthogonal to a dish in contact with the bottom of the dish. A 1 V differential was applied between the rods, and electric field values were extracted in the plane 5 µm above the surface of the dish (Figure 8). 

The same calculations were performed for the electrode array that was used in strobe imaging experiments, with the only difference being that the rods were spaced 2 mm apart (2.5 mm center-to center). The electric field non-uniformity at the cell location (within a 100-µm radius from the geometrical center between the rods) was less than ±2%.

For all other experiments in individual cells exposed to pulses within the microscope’s field of vision, the electric field at the cell location was calculated similarly to what was reported previously [32,46,66]. In brief, two parallel metal rods, 100 µm in diameter, were inserted 3 mm deep into a 1.4 S/m solution at a 30° angle to the glass coverslip with cells. The rod tips were positioned 50 µm above the surface of the glass. For all experiments, the electric field simulated at 1 V was then multiplied by the applied experimental voltage to yield the electric field values. 

### 4.9. Statistical Analysis

Grapher 16 (Golden Software) was used for graph preparation and data fitting. A two-sided Student’s t-test was used to determine significance, with *p* < 0.05 considered statistically significant. Error bars are the standard error of the mean, unless labeled differently in the graph. With many datapoints on each graph, we avoided using special symbols to preserve the clarity of graphs and noted the significance values in text only. In graphs, the statistical significance can be estimated from the gap between the error bars of the compared groups: A gap exceeding the length of the error bars indicates a significant difference at *p* ≤ 0.05 [67]. 

## Data Availability

The data presented in this study are available on request from the corresponding author.
